# The role of Mesothelin signaling in Portal Fibroblasts in the pathogenesis of cholestatic liver fibrosis

**DOI:** 10.3389/fmolb.2021.790032

**Published:** 2021-12-13

**Authors:** Hiroaki Fuji, Grant Miller, Takahiro Nishio, Yukinori Koyama, Kevin Lam, Vivian Zhang, Rohit Loomba, David Brenner, Tatiana Kisseleva

**Affiliations:** ^1^ Department of Medicine, University of California San Diego, La Jolla, CA, United States; ^2^ Department of Surgery, University of California San Diego, La Jolla, CA, United States; ^3^ Department of Surgery, Graduate School of Medicine, Kyoto University, Kyoto, Japan

**Keywords:** cholestatic liver fibrosis, activated portal fibroblasts, mesothelin (MSLN), mucin 16 (MUC16), thymocyte differentiation antigen 1 (Thy-1)

## Abstract

Liver fibrosis develops in response to chronic toxic or cholestatic injury, and is characterized by apoptosis of damaged hepatocytes, development of inflammatory responses, and activation of Collagen Type I producing myofibroblasts that make liver fibrotic. Two major cell types, Hepatic Stellate Cells (HSCs) and Portal Fibroblasts (PFs) are the major source of hepatic myofibroblasts. Hepatotoxic liver injury activates Hepatic Stellate Cells (aHSCs) to become myofibroblasts, while cholestatic liver injury activates both aHSCs and Portal Fibroblasts (aPFs). aPFs comprise the major population of myofibroblasts at the onset of cholestatic injury, while aHSCs are increasingly activated with fibrosis progression. Here we summarize our current understanding of the role of aPFs in the pathogenesis of cholestatic fibrosis, their unique features, and outline the potential mechanism of targeting aPFs in fibrotic liver.

## Introduction

Hepatic fibrosis is the outcome of chronic liver diseases, including cholestatic liver disease (primary sclerosing cholangitis (PSC), primary biliary cirrhosis (PBC), and secondary biliary cirrhosis (SBC)) (Lazaridis and LaRusso, 2016) and toxic liver injury (hepatitis B virus (HBV), hepatitis C virus (HCV), alcoholic liver disease and non-alcoholic steatohepatitis (NASH)) (Friedman, 2008; Dranoff and Wells, 2010). It is characterized by extensive deposition of extracellular matrix (ECM). Activated hepatic myofibroblasts, which are absent in the healthy liver, are the major source Collagen Type I which form the fibrous scar (Friedman, 2008). Hepatic stellate cells (HSCs) and portal fibroblasts (PFs) are believed to serve as the major source of the fibrous scar in the injured liver (Bataller and Brenner, 2005).

Cholestatic fibrosis is caused by chronic cholestatic injury (Lazaridis and LaRusso, 2016), hepatocyte apoptosis, ductular proliferation, inflammation, and activation of myofibroblasts. Both activated PFs (aPFs) and activated HSCs (aHSCs) (Dranoff and Wells, 2010) can produce myofibroblasts that drive cholestatic fibrosis. Despite extensive studies, the origin and contribution of hepatic myofibroblasts to cholestatic fibrosis remains controversial. Several studies in humans and experimental models of cholestatic fibrosis implicated aPFs in the pathogenesis of cholestatic fibrosis, suggesting that aPFs might serve as the primary targets for anti-fibrotic therapy (Dranoff and Wells, 2010; Wells, 2014). In support, aPFs contribute to the fibroproliferative responses in patients with primary and secondary biliary cirrhosis (PSC and SBC), but not in patients with toxic liver fibrosis such as HBV/HCV (Koyama et al., 2017).

Under the physiological conditions, PFs comprise a small population of cells that surround the portal vein to maintain integrity of the portal tract (Dranoff and Wells, 2010). Cholestatic (but not toxic) injury (Desmoulière et al., 1997) causes their proliferation and differentiation into Collagen Type I-producing myofibroblasts( (Dranoff and Wells, 2010), (Desmoulière et al., 1997), (Yata et al., 2003)), suggesting that aPFs are the “first responders” to the cholestasis-induced fibrogenic liver injury. Using the reporter Col-GFP mice (in which Collagen-1α(I) promoter drives expression of the GFP reporter gene in real time), aPFs were shown to comprise 70% of myofibroblasts at the onset of cholestatic fibrosis caused by the obstruction of the common bile duct (BDL), that mimics mechanical bile duct occlusion by liver stones or tumor mass. Similar results were obtained using another model of cholestatic injury, Mdr2^-/-^ mice (Smit et al., 1993) (deficient for canalicular phospholipid flippase, Mdr2/Abcb4), which develop disruption of bile duct tight junctions and basal membranes, causing bile leakage, and periportal cholestatic fibrosis (Smit et al., 1993) that resembles PSC (Fickert et al., 2002; Fickert et al., 2004; Popov et al., 2005; Fickert et al., 2009; Baghdasaryan et al., 2010; Mair et al., 2010), and mimics MDR2 deficiency in patients (Jacquemin, 2001; Fickert et al., 2004; Popov et al., 2005). Moreover, cholestasis-activated aHSCs share more resemblance with aPFs than with CCl_4_-activated aHSCs, suggesting that fibrogenic responses caused by cholestatic fibrosis differ significantly from those induced by toxic injury, and therefore the mechanism of the cholestatic fibrosis progression should be studied in further detail (Iwaisako et al., 2014).

The contribution of aPFs to liver fibrosis of different etiologies remains not well understood, mainly because of difficulties with the isolation of PFs and myofibroblasts. The most widely used method of aPF isolation is based on enzymatic digestion followed by size selection (Wen et al., 2012), as well as cell outgrowth from dissected bile and enzymatically digested liver segments(Uchio et al., 2002), (Kruglov et al., 2002). aPFs are identified by expression of Elastin, Col1a1, and other fibrogenic genes. Expression of specific markers such as Thy-1 (Knittel et al., 1999; Dudas et al., 2007; Yovchev et al., 2009; Katsumata et al., 2017), Fibulin 2 (Knittel et al., 1999), IL-6, Elastin (Goodpaster et al., 2008), the ecto-AT-Pase nucleoside triphosphate diphosphohydrolase-2 (NTPD2) (Dranoff et al., 2002), and coffilin 1 (Bosselut et al., 2010) was originally identified in aPFs, demonstrating that these cells are different from desmin, cytoglobin, GFAP, p75^NGFr^, and Vitamin A expressing HSCs (Bataller and Brenner, 2005; Dranoff and Wells, 2010; Fausther and Dranoff, 2011). The development of flow cytometry-based techniques made it possible to sort purify the population of hepatic Col-GFP^+^Thy-1^+^VitaminA^−^CD45^−^ aPFs, which can be distinguished from Col-GFP^+^Thy-1^−^VitaminA^+^ aHSCs, and identified new markers of aPFs such as Mesothelin (Msln), Muc16, CD34, Gpc3, Asporin, Bnc1 (Iwaisako et al., 2014; Nishio et al., 2021). Moreover, Msln was shown to critically regulate fibrogenic activation and proliferation of aPFs in response to cholestatic injury. This review will summarize the potential role of Msln-Thy-1 and Muc16 signaling in the activation of aPFs in experimental models of cholestatic fibrosis, and discuss the emerging strategies to target aPFs to treat cholestatic liver fibrosis.

## Cholestatic Liver Fibrosis

The etiology of cholestatic injury differs considerably from toxic liver injury. Cholestatic injury results from genetic defects or mechanical injury of the bile ducts, causing impaired hepatobiliary production and excretion of bile, accumulation of bile and liver tissue damage, apoptosis and proliferation of mature cholangiocytes and hepatocytes, inflammation, and biliary fibrosis (Fickert et al., 2009; Vavassori et al., 2009; Wagner et al., 2010). Several experimental models are routinely used to dissect the mechanism of cholestatic fibrosis, such as Mdr2^-/-^ mice (Smit et al., 1993) and BDL. Despite different etiologies, these models exhibit common pathophysiological features. Reversal of the etiological cause of cholestasis may result in regression of liver fibrosis.

## Activated Portal Fibroblasts Play a Key Role in Cholestatic Liver Fibrosis

Activation of fibrogenic Collagen Type I producing myofibroblasts is the key event leading to the progression of cholestatic fibrosis. Myofibroblasts are characterized by a spindle or stellate shape and expression of abundant intracellular proteins (vimentin, α-smooth muscle actin (α-SMA), non-muscle myosin) (Eyden, 2008), rough endoplasmic reticulum (rER) and a Golgi apparatus producing collagen (Gabbiani et al., 1971; Majno et al., 1971; Schürch et al., 1998; Eyden, 2008).

### The Origin of Myofibroblasts in Cholestatic Liver Fibrosis

**FIGURE 1:**
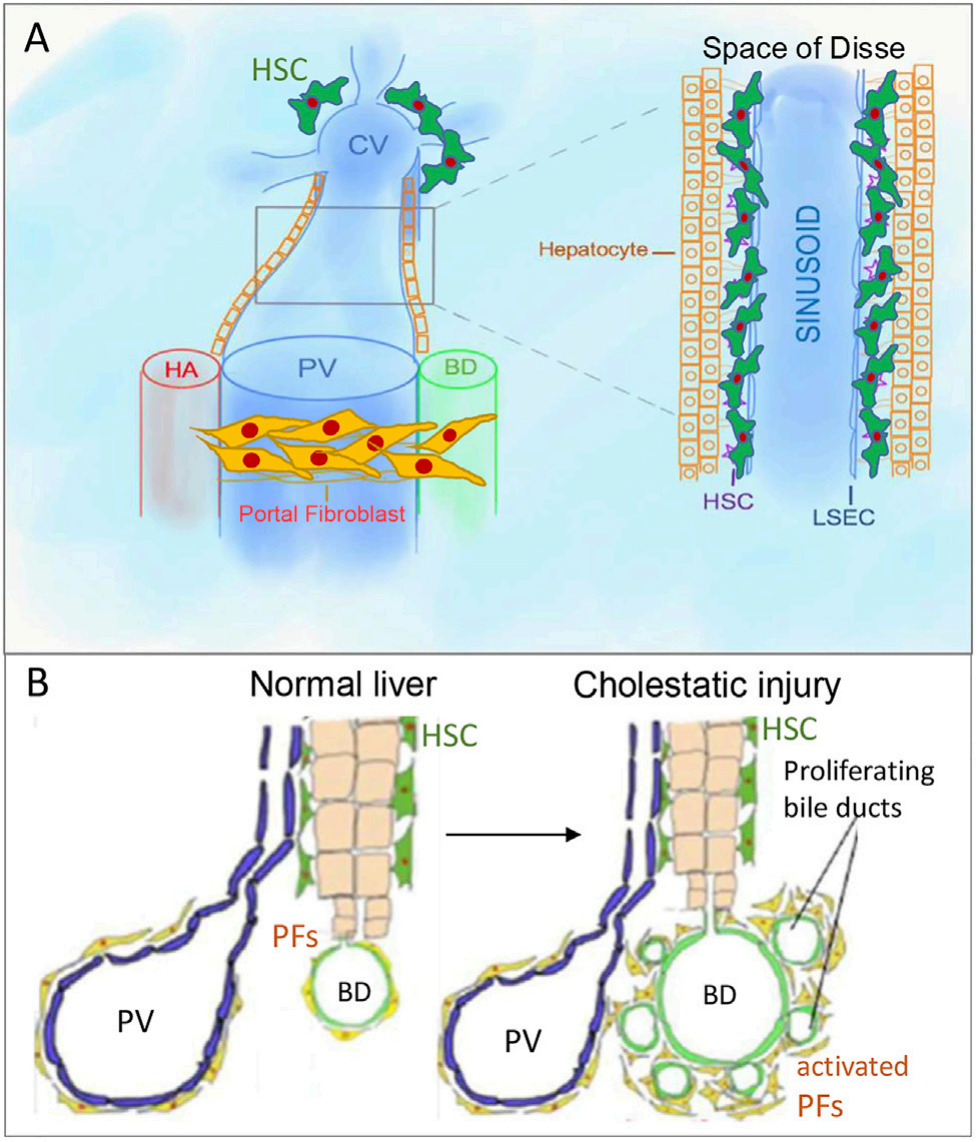
Portal fibroblasts/myofibroblasts (aPFs/MFs) and hepatic stellate cells (HSCs). **(A)**. PFs are located around portal triads, while HSCs are located in the space of Disse, which is between sinusoidal endothelial cells and hepatocytes cluster. **(B)**. Bile ducts proliferate in response to bile duct ligation, known as “ductular reaction.” PV, portal vein, CV, central vein, HA, hepatic artery, BD, bile duct.

The cell that secretes the fibrillary collagens leading to cholestatic fibrosis has a long and controversial history (Dranoff and Wells, 2010; Mederacke et al., 2013). Due to lineage tracing studies by our lab (Iwaisako et al., 2014; Koyama et al., 2017) and others (Asahina et al., 2009; Asahina et al., 2011), there is a clear consensus that endogenous mesenchymal cells activate to become myofibroblasts that secrete the fibrous scar proteins. Fate mapping studies have also demonstrated that epithelial mesenchymal transition (EMT) (Scholten et al., 2010; Taura et al., 2010; Chu et al., 2011), or recruited fibrocytes (Kisseleva et al., 2006; Scholten et al., 2011; Iwaisako et al., 2014) are not major contributors to the myofibroblast population. In turn, two hepatic mesenchymal cells become myofibroblasts depending on the fibrotic stimulus (Iwaisako et al., 2014). Hepatotoxic liver injury activates HSCs to become myofibroblasts, while cholestatic liver injury activates both HSCs and aPFs (Dranoff et al., 2002; Kruglov et al., 2002; Wen et al., 2012). aPFs comprise 70% of myofibroblasts at the onset of bile duct ligation (BDL)-induced injury, while aHSCs are increasingly activated with fibrosis progression (Iwaisako et al., 2014; Karin et al., 2016) (Figures 1A,B).

### Hepatic Stellate Cells

Under physiological conditions, quiescent HSCs express desmin, neural markers (glial fibrillar acidic protein (GFAP), synaptophysin (Bataller and Brenner, 2005), NGF receptor p75 (Sachs et al., 2007; Kendall et al., 2009)), and Vitamin A droplets( (Iredale, 2007; Geerts, 2001; Senoo et al., 2007)) and reside in the space of Disse (Figure 1A), but in response to injury differentiate into aHSCs/myofibroblasts expressing vimentin, and collagens (Kisseleva and Brenner, 2006; Fallowfield et al., 2007).

### Portal Fibroblasts

In normal liver, portal fibroblasts (PFs) comprise a small population of “periductular mesenchymal cells” that surround the portal vein and maintain integrity of the portal tract (Figure 1B) (Desmoulière, 2007; Dranoff and Wells, 2010; Wells, 2014). In response to cholestatic injury (but not toxic carbon tetrachloride (CCl_4_)-induced injury) (Desmoulière et al., 1997), activated portal fibroblasts (aPFs) proliferate, upregulate Col1a1, TIMP1, Spp1, TGFβRI, TGFβ2, and secrete extracellular matrix (ECM) (Desmoulière et al., 1997; Yata et al., 2003; Dranoff and Wells, 2010). aPFs are identified by expression of Thy-1( (Knittel et al., 1999; Dudas et al., 2007; Yovchev et al., 2009)), Fibulin 2 (Knittel et al., 1999), Elastin (Goodpaster et al., 2008), NTPD2 (Dranoff et al., 2002), coffilin 1 (Bosselut et al., 2010), Msln, Muc16, Apsorin, Bnc1, Upk1β, Calca, Gpc3 ((Koyama et al., 2017), (Iwaisako et al., 2014)). We have recently demonstrated that Msln, Muc16 (Koyama et al., 2017), and Thy-1 (Katsumata et al., 2017) play a critical role in regulation of aPF biology.

## Unique Features of Activated Portal Fibroblasts

Based on gene expression profiling, BDL-activated aPFs expressed genes that distinguish them from CCl_4_-activated aHSCs, and were identified as “signature genes” for aPFs. In concordance with previous studies (Kawada et al., 2001; Bosselut et al., 2010), aPF signature genes included Thy-1, Elastin, Gremlin 1, Fibulin 2, and NTPD2 (Dranoff and Wells, 2010; Forbes and Parola, 2011), but also the newly identified genes, Msln, and Muc 16, Calca, Upk1β, Bnc1 and others. Human MSLN^+^THY1^+^αSMA^+^ aPFs also express aPF-specific markers (UPK1b, CD200, EMILIN2, BNC1, ASPN, GPC3, and GREM1) similar to that observed in mouse aPFs, suggesting that upregulation of these specific genes in activated PFs is preserved among species. Some of these genes Msln, Calca, Upk1β, Bnc1 were reported as signature genes of murine hepatic mesothelial (Onitsuka et al., 2010) and epicardial cells (Bochmann et al., 2010), supporting the theory that PFs originate from mesothelial cells (Asahina et al., 2009; Asahina, 2012). Expression of Msln and Muc16 is detected in Thy-1^+^ aPFs but not in qHSCs, aHSCs, endothelial cells (EC), Kupffer cells (KC), or cholangiocytes. The fact that expression of Msln was detected only in isolated aPFs but not in other liver fractions suggests (Iwaisako et al., 2014) that Msln expression might be important for aPF biology.

## Historical Characterization of MSLN, CA125 and Thy-1

### Mesothelin

Msln (Chang and Pastan, 1996) is Glycosylphosphatidyl inositol (GPI)-linked membrane-anchored protein (71 kDa, Msln precursor). Originally, MSLN was identified as a tumor marker. Human MSLN is strongly upregulated in several human malignancies, including mesotheliomas and ovarian cancer, and is a target for anti-cancer therapy. Anti-MSLN Abs have been generated and are being tested in clinical trials in patients with ovarian cancer.

### Mucin 16

Muc16 is the murine analogue of human CA125 (McMullen et al., 2005). Studies of patients with ovarian cancer have identified the cancer antigen CA125 as a Msln ligand (Pastan and Hassan, 2014), which is widely used as a diagnostic marker (with the exception of liver and lung cirrhosis which are considered as “false positives” (Scholler and Urban, 2007). CA125 is a member of the membrane-tethered family of mucins, which contains a transmembrane domain with a short cytoplasmic domain, and highly glycosylated at N-terminus (Pastan and Hassan, 2014) and is a MSLN ligand (Gubbels et al., 2006; Kaneko et al., 2009).

### Msln-Muc16 Signaling in Cancer Cells

Since its discovery in 1992 as a cancer antigen, the mechanism of human MSLN signaling remains unresolved. Until recently, CA125 (mouse Muc16) remained the only known ligand of MSLN that activates Src/Akt signaling in cancer cells. In cancer cells MSLN-Muc16 signaling increases cancer cell proliferation and metastasis. Msln-mediated secretion of MMP-7 in MUC16-expressing cancer cells occurs via a p38 MAPK-dependent pathway. Depletion of MMP-7 or inhibition of p38 activity abolishes MSLN-mediated cancer cell motility and invasion. Knockdown of Msln suppresses tumor invasiveness in xenograft models in mice (He et al., 2017). Although, Msln^-/-^ and Muc16^-/-^ mice have a normal phenotype until injury or stress (Bera and Pastan, 2000; McMullen et al., 2005), when subjected to experimental model of liver cancer, Msln-knockout mice developed a defect in activation of cancer associated myofibroblasts (Zhang et al., 2011).

### Msln as a Mesothelial Marker

Expression of Msln is not restricted to cancer cells or cancer-associated myofibroblasts but is also induced in aPFs. Msln also serves as a mesothelial cell marker (Pastan and Hassan, 2014). Msln is highly expressed during embryonic development (Majno et al., 1971; Iwaisako et al., 2014) but minimally expressed in adulthood (Pastan and Hassan, 2014). In adult mice and humans, Msln-expressing stem-like cells reside in the mesothelial layer lining of parenchymal organs and serosal cavities (Bera and Pastan, 2000) in a dormant state, and do not proliferate until injury or stress, and have a capability to give rise to the mesenchymal and mesothelial cells, as well as fibroblasts.

### Thy-1 (CD90, Cluster of Differentiation 90)

Thy-1 is a 25–37 kDa heavily N-glycosylated (GPI)-linked cell surface protein (Nosten-Bertrand et al., 1996), with a single V-like immunoglobulin domain, originally discovered as a thymocyte antigen.Thy-1 is a GPI-anchored protein (like Msln) (Nosten-Bertrand et al., 1996) expressed in fibroblasts, T cells and neurons, and considered to be a specific marker for these cell types. Thy-1 was implicated in inhibition of TGFβ1 responses in tissue fibroblasts. Studies of lung fibroblasts have demonstrated that deletion of Thy-1 in mice exacerbated bleomycin-induced lung fibrosis (Ramírez et al., 2011). Thy-1 was shown to signal via the Src-family kinase (SFK) and focal adhesion kinase (FAK) pathways (Bradley et al., 2009) to prevent TGFβ1-induced fibroblast activation (Koyama et al., 2017) and inhibition of extracellular activation of tissue-associated latent TGF-β1 via interaction with αν-β5 integrins at the cell surface (Zhou et al., 2010), suggesting that Thy-1 can function as a mechanosensor (Fiore et al., 2015). Thy-1 expression in murine lung fibroblasts is decreased with fibrosis progression (McIntosh et al., 1994; Hagood et al., 1999; Hagood et al., 2005; Sanders et al., 2007; Zhou et al., 2010; Sueblinvong et al., 2012). Thy-1 also modulates lipid raft-associated signaling promoting fibroblast adhesion and limiting migration (Bradley et al., 2009).

### Thy-1 in Fibroblasts was Linked to Fibrosis

Thy-1 is silenced in lesional fibroblasts in IPF (Idiopathic Pulmonary Fibrosis), and its expression in murine lung fibroblasts is decreased with progression of experimental bleomycin induced lung fibrosis (Hagood et al., 2005; Sueblinvong et al., 2012). Thy-1 acts as a fibrosis suppressor which prevents differentiation of lung fibroblasts into myofibroblasts (including Collagen Type I expression, cytokine and growth factor expression, migration, and cell survival). Upon activation, lung myofibroblasts upregulate TGFβ1-responsive genes (Activin and PAI-1) but downregulate expression of Thy-1 (McIntosh et al., 1994; Hagood et al., 1999; Hagood et al., 2005; Sanders et al., 2007; Zhou et al., 2010). Deletion of Thy-1 exacerbates development of cholestatic fibrosis in mice (Koyama et al., 2017; Nishio et al., 2021).

## Msln Signaling Plays a Critical Role in Activation and Proliferation of Activated Portal Fibroblasts

The molecular mechanisms underlying Msln signaling in experimental models of cholestatic fibrosis have been evaluated, and demonstrated that in addition to Muc16, Msln can also bind to Thy1 in aPFs and form a signaling Msln-Muc16-Thy-1 complex that regulates fibrogenic activation and proliferation of aPFs.

### Msln^-/-^ and Muc16^-/-^ Mice are Protected From Cholestatic Liver Fibrosis

Although, Msln^-/-^, Muc16^-/-^, and Thy-1^-/-^ mice exhibit no obvious abnormalities under physiological conditions (Bera and Pastan, 2000; McMullen et al., 2005), these molecules play a critical role in the pathogenesis of cholestatic fibrosis. Thus, cholestatic fibrosis (caused by BDL or Mdr2 deficiency) was strongly attenuated by ≈ 50% in Msln knockout mice (Msln^-/-^ mice). *In vitro* analysis revealed that Msln regulates TGFβ1-inducible activation of the wild type aPFs, and facilitates their FGF-FGFRI-Act-mediated aPF proliferation (via inhibition of FGFRI turnover and re-expression). Similarly, deletion of Muc16 (the binding partner of Msln and potentially the only transmembrane signaling molecule in this complex) also attenuates development of cholestatic fibrosis, outlining the importance of Msln-Muc16 interaction. Moreover, ductular proliferation was reduced in cholestasis-injured Msln^-/-^Mdr2^-/-^ mice and Muc16^-/-^Mdr2^-/-^ mice, suggesting that aPF activation regulates cholangiocyte proliferation.

Thy-1^-/-^ mice are more susceptible to cholestatic fibrosis. Studies of the experimental models of cholestatic fibrosis in wild type, Msln^-/-^ mice, Muc16^-/-^ mice, and Thy-1^-/-^ mice have demonstrated that Msln and Muc16 play pro-fibrogenic roles in aPF activation, while Thy-1 exhibits anti-fibrogenic properties. Consistently, cholestatic fibrosis is exacerbated in Thy-1^-/-^ mice. These findings were supported by *in vitro* comparison of primary isolated mouse wild type, Msln^-/-^, Muc16^-/-^, and Thy-1^-/-^ aPFs. In resting aPFs, Thy-1 directly binds to TGFβRI and blocks TGFβ1 binding to TGFβRI, thereby preventing TGFβ1 signaling.

## Msln, Muc16 and Thy-1 Regulate Non-canonical TGFβ1-TGFβRI Signaling in Cholestasis-Activated Portal Fibroblasts

### Formation of Thy-1-TGFβRI in Resting aPFs Prevents TGFβ1 Signaling

**FIGURE 2:**
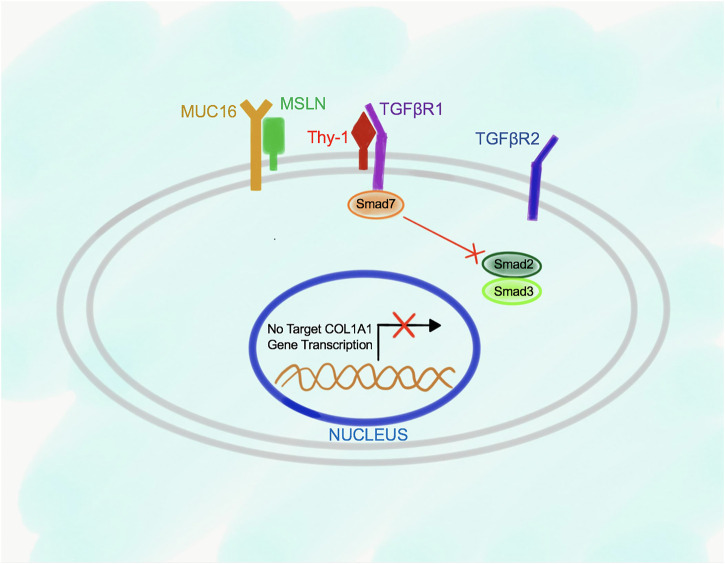
Proposed model of Msln-Muc16 and Thy-1-TGFβRI binding in resting wild type aPFs. Msln and Muc16 form a complex in resting aPFs. Thy-1 and TGFβRI form a complex. Binding of Thy-1 to TGFβRI prevents TGFβ1 signaling, and retains Smad7 at the C-terminus of the TGFβRI.

The relationship between Msln, Muc16, Thy-1, and TGFβRI receptors in the wild type and Msln^-/-^ aPFs was established using immunoprecipitations (IPs) with specific antibodies against each molecule. Although not quantitative, this technique allowed to determine the dynamic changes in the protein binding between Msln, Muc16 and Thy-1 in the resting wild type aPFs and in response to TGFβ1 stimulation. We have demonstrated that in resting (serum starved) aPFs Thy-1 makes an inhibitory complex with TGFβRI receptor thereby preventing TGFβ1 binding to the N-terminus of TGFβRI. Thy-1 also binds to Muc16 but has minimal interaction with Msln (Figure 2). Meanwhile, Msln forms a strong complex with Muc16, suggesting that Muc16 transmits intracellular signals from Msln-Muc16 complex. TGFβ1 signaling is further inhibited by Smad7 (transcription factor implicated in suppression of TGFβ1 signaling), which is bound to the C-terminus of the TGFβRI and prevents Smad2/3 docking and phosphorylation on TGFβRI.

### TGFβ1 Signaling in aPFs Promotes Disruption of Thy-1-TGFβRI Complex and Formation of Msln-Muc16-Thy-1 Complex

**FIGURE 3:**
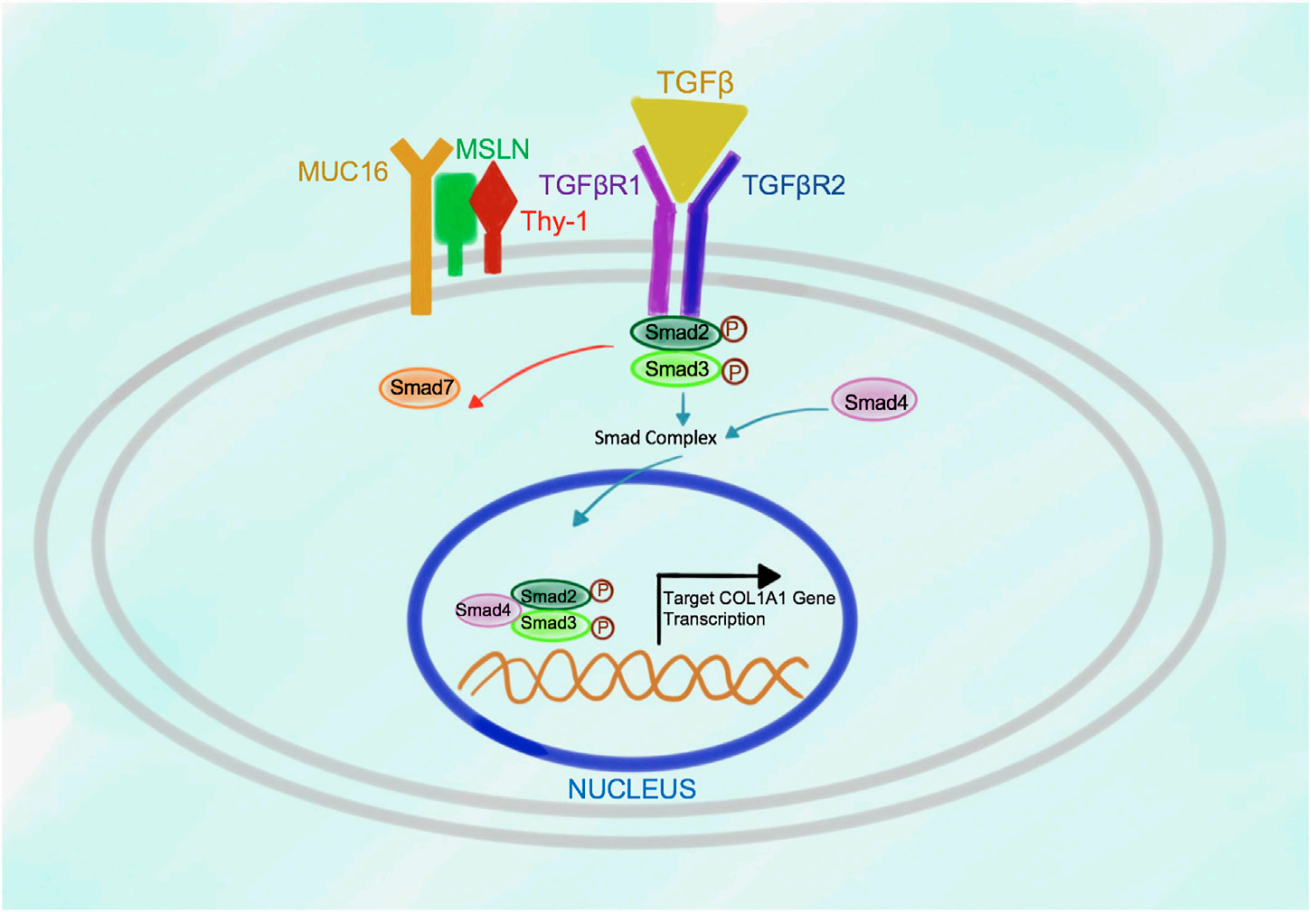
Proposed model of Msln-Muc16-Thy-1 binding in TGFβ1-stimulated wild type aPFs. In response to TGFβ1 signaling Msln-Muc16 complex binds to Thy-1 causing dissociation of Thy-1 from TGFβRI. TGFβ1 binding to TGFβRI and TGFβR2 causes receptor crosslinking, docking of Smad2/3 to the receptors. Upon Smad2/3 phosphorylation, *p*-Smad2/3 dissociates from the receptors, forms a complex with Smad4, and translocates to the nucleus where it initiates transcription of target genes.

In turn, in response to stimulation of the wild type aPFs with TGFβ1, binding of TGFβ1 to TGFβRI strongly increases the affinity of Msln to Thy-1 causing dissociation of Thy-1 from TGFβRI (Figure 3). Formation of Msln-Muc16-Thy-1 complex results in disruption of Thy-1-TGFβRI interaction and removal of Thy-1 from TGFβRI. TGFβI binds to TGFβRI and TGFβRII, causing dissociation of Smad7 from TGFβRI and subsequent binding of Smad2/3 to the C-terminus of TGFβRI where these transcription factors are phosphorylated and activated. Phosphorylated Smad2/3 are released from TGFβRI into the cytoplasm where they form a complex with Smad4. *p*-Smad2/3-Smad4 are translocated to the nucleus, where they bind to the DNA and initiate transcription of the fibrogenic genes, including Collagen Type I.

### TGFβ1-TGFβRI Signaling is Suppressed in Msln-Deficient aPFs

**FIGURE 4:**
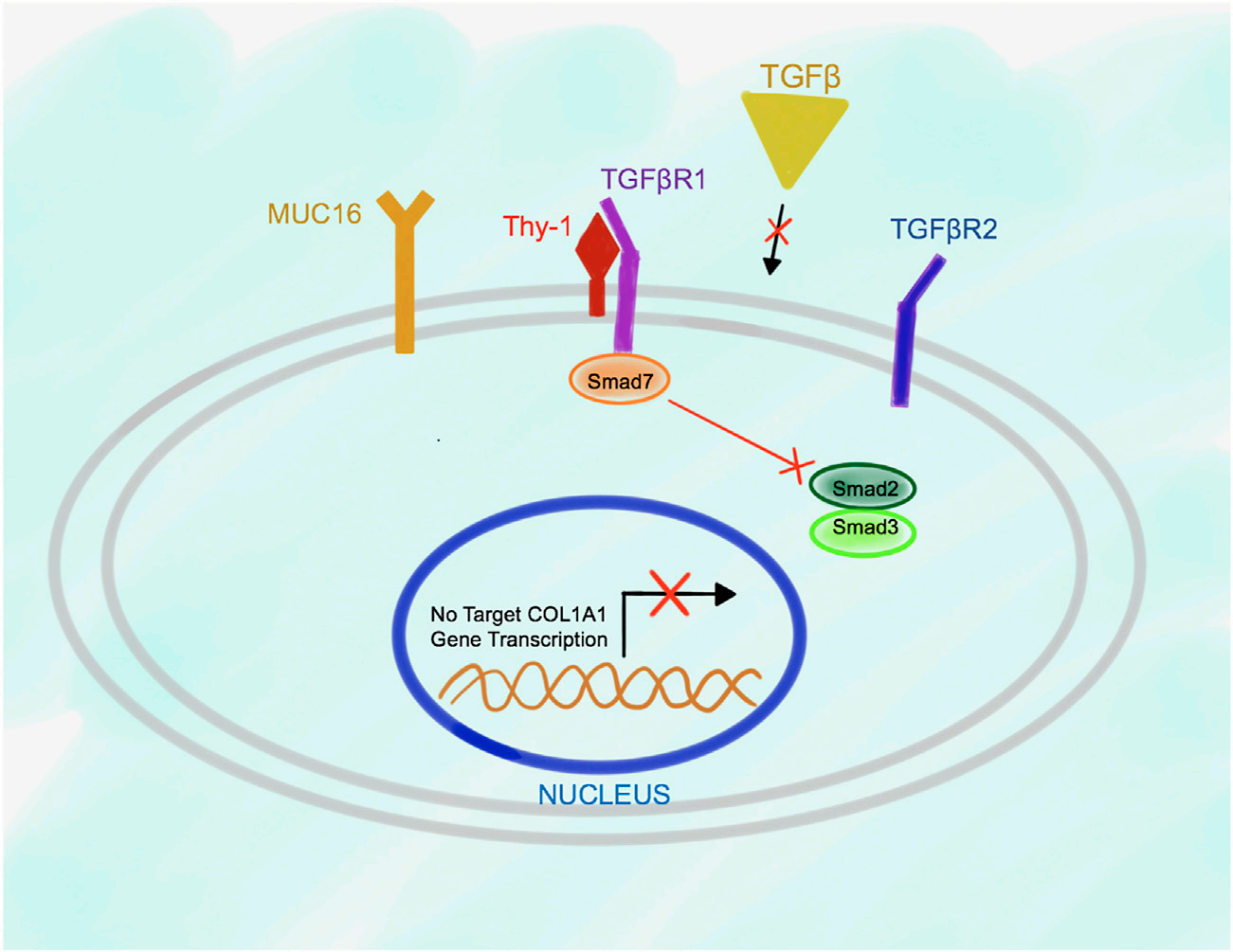
Proposed model of Msln-Muc16-Thy-1-TGFβRI signaling in Msln^-/-^ aPFs. TGFβ1 signaling is impaired in Msln^-/-^ aPFs because Thy-1 forms a stable complex with TGFβRI, which hinders TGFβ1 binding to TGFβRI and TGFβR2. Smad7 is bound to the cytoplasmic tail of TGFβRI, thereby preventing docking and phosphorylation of Smad2/3.

Deletion of Msln results in suppression of TGFβ1-TGFβRI signaling in aPFs due to increased Thy-1 expression, and higher affinity of Thy-1 binding to TGFβRI (than in the wild type aPFs), indicating that Thy-1 serves as an inhibitory molecule for the TGFβ1 signaling in aPFs (Figure 4). Under these circumstances, Smad7 is constitutively bound to the C-terminus of TGFβRI, suggesting that lack of Msln (or increased Thy-1-TGFβ1RI binding) promotes Smad7 docking to the cytoplasmic C-terminus of the TGFβRI. As a result, activation and phosphorylation of Smad2/3 is reduced in Msln^-/-^ aPFs; production of fibrogenic genes and Collagen Type I is suppressed.

### TGFβ1-TGFβRI Signaling is Accelerated in Thy-1-Deficient aPFs

Moreover, deletion of Thy-1 in aPFs results in strong overexpression of Msln in Thy-1^-/-^ aPFs, indicating that Thy-1 is a critical regulator of Msln. Indeed, Thy-1^-/-^ aPFs produce more Col1a1 mRNA in response to TGFβ1 stimulation, and this effect is associated with increased phosphorylation of Smad2/3 and expression of TGFβRI, while binding of Smad7 to TGFβRI is decreased in Thy-1^-/-^ aPFs. We speculate that genetic deletion of **Thy-1** gene results in exacerbation of Msln signaling caused by the compensatory overexpression of Msln and its target genes. It remains unknown if this effect can be solely attributed to the strong upregulation of Msln (≈7 fold over the wild type aPFs) in Thy-1^-/-^ aPFs, and/or the loss of Thy-1 functions (such as binding to TGFβRI suppression of Msln expression). Since Thy-1 is a GPI-linked protein, Thy-1 might bind to another transmembrane signaling receptor (distinct from Muc16), or utilize the lipid rafts protein signaling to mediate its function.

### TGFβ1-TGFβRI Signaling is Not Affected in Double Knockout Msln^-/-^Thy-1^-/-^ aPFs

Generation of double knockout Msln^-/-^Thy-1^-/-^ aPFs revealed that Thy-1 and Msln might regulate one signaling pathway, since simultaneous deletion of Msln and Thy-1 abolished both phenotypes, and double knockout Msln^-/-^Thy-1^-/-^ aPFs exhibited no obvious abnormalities. In support, simultaneous deletion of Msln and Thy-1 genes yielded a phenotype similar to that in the cholestasis-injured wild type mice, indicating that Msln and Thy-1 might regulate opposing functions within the same signaling pathway. These new findings suggest that Msln-Muc16-Thy-1 signaling plays an important role in the regulation of TGFβ1-TGFβRI signaling in cholestasis-activated aPFs.

## Msln as a Target for Anti-fibrotic Therapy

### Thy-1^+^ and Msln^+^ aPFs are Expressed in Livers of Patients With Cholestatic Liver injury but not Toxic HCV Fibrosis

When the composition of myofibroblasts was analyzed in livers of patients with liver fibrosis, the expression of MSLN and THY-1 was upregulated in livers of PSC patients, patients with biliary atresia, and biliary cirrhosis (but not in livers of patients with HCV liver fibrosis). Expression of human THY-1 and MSLN correlated with the stage of cholestatic fibrosis, suggesting that MSLN^+^ aPFs can be a novel target for anti-fibrotic therapy. Msln is widely expressed in embryonic mesothelium during mammalian development (Akira et al., 2006). In turn, Msln is minimally expressed in adult mice and healthy humans under physiological conditions. Upregulation of MSLN in adult humans is associated with cancer, and was recently linked to the development of cholestatic fibrosis (Pastan and Hassan, 2014).

### Potential Strategies to Target aPFs

Historically high expression of MSLN was linked to increased tumor proliferation/invasion. Therefore, Msln serves as a target for anti-cancer therapy. We tested if targeting MSLN could also be beneficial for halting cholestatic fibrosis. Three classes of potential Msln inhibitors have been generated and potentially used to block MSLN-MUC16-THY-1 signaling pathway in patients: anti-human MSLN Ab-immunotoxin (that causes death of human MSLN^+^ cancer cells) (Hassan et al., 2007); anti-MSLN blocking Abs can potentially suppress growth and proliferation of aPFs (Onda et al., 2005); or recombinant human soluble THY1 (hsTHY1, that neutralize reactivity to αν-β5 integrins, and bind to TGFβRI to prevent MSLN signaling) (Tan et al., 2019). These tools can potentially be used in patients with cholestatic fibrosis.

### Immunotherapy to Target Cancer Cells

Immunotherapy-based strategy to target human cancer cells was developed by Dr. Pastan and colleagues, pioneers in the field of cancer research. Specifically, much progress has been made with immunotherapy-based therapeutics of human MSLN^+^ malignancies. MSLN is differentially expressed between normal and cancer cells, thus making it a strong candidate for anti-cancer therapy with recombinant immunotoxins (RITs) (Liu et al., 2012). Several generations of immunotoxins, such as SS1P and LMB100, were engineered by conjugation of anti-human MSLN SS1 Ab (Hassan et al., 2007; Hassan et al., 2014) to PE38 (truncated *Pseudomo-nas* exotoxin, that causes cellular apoptosis) (Hassan et al., 2000), and successfully tested in clinical trials in patients with mesothelioma, ovarian cancer and pancreatic cancer (Liu et al., 2012; Kreitman et al., 2009; Chowdhury and Pastan, 1999; Alewine et al., 2014))(https://clinicaltrials.gov/ct2/show/NCT02810418) (Hassan et al., 2007; Hassan et al., 2014; Kreitman et al., 2009). In detail, SS1(dsFv)PE38 (SS1P) is a RIT that consists of a modified bacterial toxin *Pseudomonas* exotoxin A (PE38) that is bound to the anti-MSLN Ab (SS1(dsFv)) directed against the MSLN antigen expressed on the surface of the target cells (Chowdhury and Pastan, 1999). Once bound to MSLN, the entire RIT molecule is internalized, leading to the release of PE38 into the cytosol and cellular apoptosis via inactivation of ADP-ribosylation/elongation factor 2 pathway (Hassan et al., 2000; Pastan et al., 2007).

### Targeting Msln^+^ aPFs With immunotoxins as Potential Strategy for Treatment of Cholestatic Fibrosis

The question remains if a similar strategy can be used to ablate aPFs to eliminate the source of Collagen Type I. Based on our previous findings in mice, genetic ablation of aPFs (using overexpression of Diphtheria Toxin α, DTA) causes aPF apoptosis without causing structural liver damage, and attenuates development of cholestatic fibrosis in BDL-injured mice (Koyama et al., 2017), outlining that immunotoxin-based ablation of human aPFs may become a novel strategy for treatment of PSC patients. In accord, SV40-Large SS1P and LMB100 immunotoxins (Hassan et al., 2007) can successfully kill human primary cultured aPFs *in vitro,* but also *in vivo* in the xenograft mice, generated by adoptive transplantation of human primary aPFs into the livers of adult immunodeficient Rag2^-/-^γc^-/-^ mice (Nishio et al., 2021). Generation of “human aPF xenograft” Rag2^-/-^γc^-/-^ mice is novel, and might serve as a useful model to study *in vivo* the variability of patient-specific responses of human aPFs (fibrogenic activation/proliferation) to specific MSLN inhibitors (Nishio et al., 2021).

A potential drawback is that repeated administration of RITs (Kreitman et al., 2009) might lead to the formation of anti-drug antibodies (ADAs) and accelerated clearance of anti-MSLN-immunotoxins (Baker et al., 2010). LMB100 was engineered to reduce immunogenicity in humans compared with SS1P (Liu et al., 2012; Alewine et al., 2014). Both immunotoxins successfully showed excellent anti-tumor activity in clinical trials in patients with mesothelioma, ovarian and pancreatic cancer (Kreitman et al., 2009; Hassan et al., 2014).

### Blocking of Msln Expression in aPFs May Attenuate Cholestatic Liver Fibrosis

Administration of blocking unconjugated anti-Msln As (Koyama et al., 2017) might also be beneficial in suppression of aPF proliferation and activation. Such strategy was explored in BDL-injured mice, and repetitive administration of Msln-blocking Abs (D233-3**,** 5ng, 10 ng, MBL Inc.; or B35 Ab, 10 ng, LSBio) was shown to inhibit aPFs and reduced cholestatic fibrosis.

### Human Soluble hsTHY-1-Fc Peptide

THY-1 exhibits anti-fibrogenic properties. Human soluble THY-1 peptide shares high similarity with mouse soluble Thy-1 and crossreacts with mouse ligands. Binding of hsTHY-1 (but not hsTHY-1-RLE with mutated integrin-binding RGD-like motif) (Tan et al., 2019) to αvβ5 integrin was shown to prevent activation of latent TGFβ1 in lung fibroblasts (Zhou et al., 2010). Based on our unpublished observation, administration of hsTHY-1 peptide (1 μg/g in PBS) suppressed BDL-induced aPF activation in BDL-injured mice and attenuated development of cholestatic fibrosis (compared to mutant hsTHY-1-RLE- or vehicle-treated mice). We can speculate that administration of hsTHY-1 also prevents TBFβ1-TGFβRI signaling.

## Conclusion

Investigation of the role of Msln, Muc16, and Thy1 in cholestatic fibrosis revealed that Msln^-/-^ mice are protected from cholestatic fibrosis caused by Mdr2 deficiency, or BDL-induced obstruction of the common bile duct. There is a growing evidence that Msln is a critical activator of aPFs. Msln expression correlates with the stage of liver fibrosis in patients with PSC. Anti-MSLN Ab-immunotoxins, developed for cancer therapy, can potentially be used to target human MSLN^+^ aPFs for treatment of cholestatic fibrosis. Overall, immunotherapy-based ablation of human aPFs might become a novel strategy for treatment of cholestatic fibrosis. It might not cure patients with cholestatic fibrosis but can decrease fibroproliferative responses to bridge PSC patients to liver transplantation, or treatment of the etiological causes.

## Abbreviations

Msln, mouse mesothelin (human counterpart MSLN); Muc16, mouse mucin 16 (human counterpart CA125); Thy1, mouse thymocyte differentiation antigen 1 (human counterpart THY1).
